# On the Dielectric Behavior of Amine and Anhydride Cured Epoxy Resins Modified Using Multi-Terminal Epoxy Functional Network Modifier

**DOI:** 10.3390/polym11081271

**Published:** 2019-07-31

**Authors:** Istebreq A. Saeedi, Thomas Andritsch, Alun S. Vaughan

**Affiliations:** Department of Electronics and Computer Science, Faculty of Engineering and Physical Sciences, University of Southampton, Southampton SO17 1BJ, UK

**Keywords:** modified resins, β relaxation, anhydride cured, gamma relaxation, functional network modifiers, molecular dynamics

## Abstract

A range of modified amine- and anhydride-cured epoxy systems based upon diglycidyl ether of bisphenol A was produced, through the systematic incorporation of moieties termed functional network modifiers (FNMs) that serve to change the network structure in controlled ways. Here, the chosen FNM was trimethylolpropane triglycidyl ether (TTE). The resulting materials were characterized by Fourier transform infrared spectroscopy, thermal analysis, dielectric spectroscopy and measurements of direct current conduction. A progressive reduction in the glass transition temperature of the modified samples was seen with increasing TTE, which is interpreted in terms of changes in the network architecture of the resin. The molecular origins of the dielectric γ and β relaxation processes are proposed. The observed increase in conduction seen exclusively with increasing TTE content in the amine-cured systems is considered in terms of the chemistry of the FNMs, variations in free volume, changes in molecular dynamics and residual unreacted groups retained from the curing reaction. Specifically, we relate the observed increase in conduction to the presence of unreacted amine groups.

## 1. Introduction

**Figure 1:**
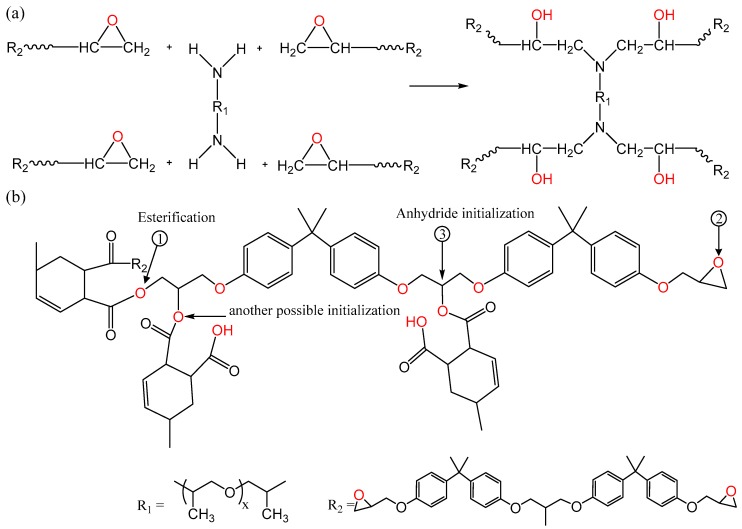
Schematic representation of the network formed through chemical reactions between a diglycidyl ethers of bisphenol A (DGEBA) epoxy resin and (**a**) a poly-ether-amine based hardener and (**b**) an anhydride hardener.

Epoxy resins are thermosetting polymers, which are widely used in diverse applications including dielectric materials, adhesives and fiber-reinforced composites. While many different classes of resins exist, such as cycloaliphatic, phenol-formaldehyde and bisphenols based epoxies [1,2,3], many commonly used resin systems are based upon diglycidyl ethers of bisphenol A (DGEBA). Although the mechanism of epoxy curing is complex, the principle chemical pathways that determine the cross-linking process have been discussed in the literature [3,4,5]. For example, when adding a hardener containing primary amines to a DGEBA resin, a primary amine reacts with an epoxide ring to form a secondary amine and a hydroxyl group. Then, the consequent secondary amine undergoes a further reaction with another epoxide group, producing an additional hydroxyl group and creating a tertiary amine. This reaction continues, in principle, until all the active groups in the hardener and/or the resin are consumed. In practice, however, vitrification means that consumption of all reactive groups is unlikely. Nevertheless, an idealized schematic diagram that illustrates the curing of an epoxy resin using an amine hardener is presented in Figure 1a.

**Figure A1:**
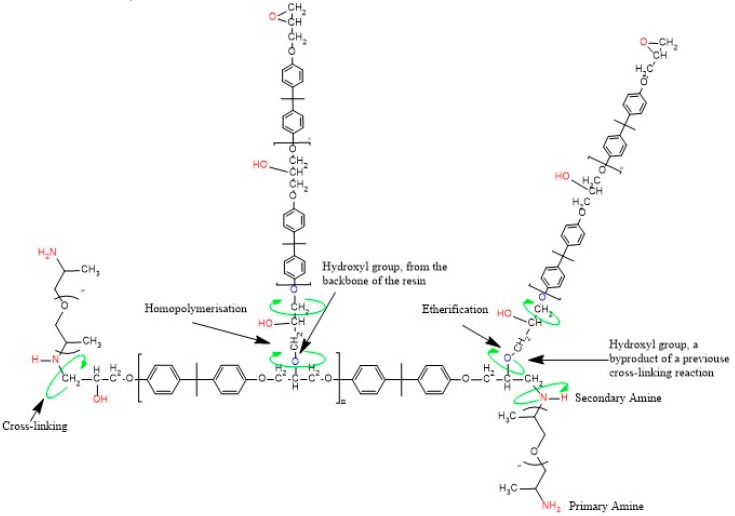
A comparison between etherification and homopolymerization for amine-cured systems.

Epoxy curing with anhydride hardeners are subject to a number of competing processes. Since the anhydride groups cannot react directly with epoxide groups, the anhydride rings first go through an initiation reaction, whereby they react with hydroxyl (OH) groups present in the system, forming a monoester containing a carboxylic acid group. The corresponding carboxylic acid intermediate then reacts with an epoxide group, producing an ester linkage and a hydroxyl; this process, termed an esterification reaction [6,7,8], is shown schematically in Figure 1b. Also, epoxide groups may react with OH groups in the system, forming ether links in a process termed etherification, albeit that if the OH group originates from the backbone of the resin, the reaction is commonly referred to as homopolymerization (Figure A1). Both etherification and homopolymerization can occur in amine and anhydride cured systems, their probability increasing with temperature [5].

**Figure 2:**
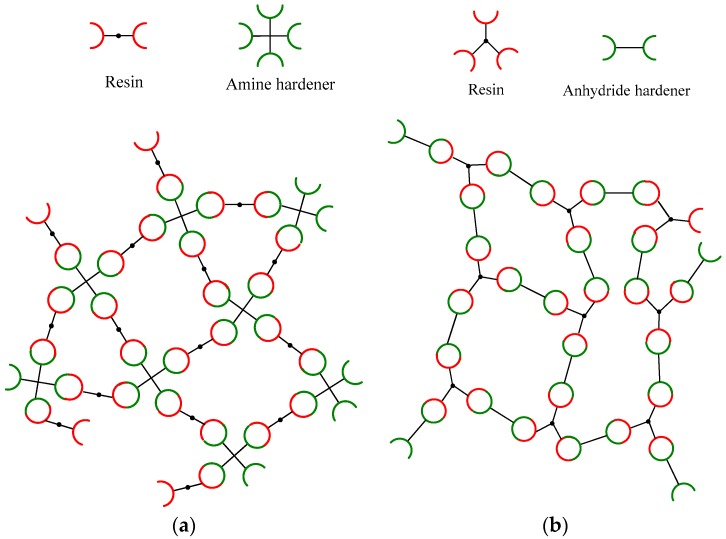
Schematic diagram showing the expected reactivity of the DGEBA resin in an (**a**) unmodified amine-cured system, (**b**) unmodified anhydride cured system. Etherification process was neglected.

As can be seen from the above account, the reactions that lead to network formation during curing of a DGEBA resin vary, depending on the curing agent used. The resin can have a different reactivity, depending on the type of hardener used. This is illustrated in Figure 2, which seeks schematically to represent the network structure produced by the various reactions shown in Figure 1 when a DGEBA resin is cured using amine and anhydride hardeners. In this Figure, the DGEBA is represented solely in terms of the dominant reactive groups present in the reactants which, when used with an amine hardener, are taken to be just the two terminal epoxide groups. However, in the case of anhydride cured systems, in addition to the two terminal epoxide groups of the resin, OH groups react in the anhydride initiation process. These OH groups are found on the backbone of the resins; ideally there is one OH group in the case of the DGEBA molecule [1]. The amine and anhydride hardeners are schematically represented in an equivalent manner as tetrafunctional (four amine hydrogens) and bifunctional (two terminal anhydride groups), respectively. For simplicity, Figure 2 assumes optimum stoichiometry and neglects processes such as etherification in amine-cured systems that are not usually favored. 

The above account demonstrates that changes in the cure chemistry affect the structure of the molecular network that forms, which influences macroscopic properties. Indeed, Carbas et al., considered the influence of curing conditions on the mechanical properties of different types of epoxy resins, where it was reported that strength and stiffness are governed by the curing and post-curing temperatures [9,10]. An additional mean of varying the mechanical properties of a given resin/hardener system is through the deliberate introduction of reactive species that modify the final network structure. So-called reactive diluents have been mainly used to increase the impact strength of epoxy resin systems, such as hydroxyl-terminated polyester [11], polyurethane-based reactive diluents [12] and liquid crystalline polyurethane-imide [13]. While such work demonstrates variations in bulk properties in response to variations in material processing or composition parameters, in general, this has involved a phenomenological approach rather than considering molecular factors. We are unaware of any previous work in which the introduction of covalently bonded functional groups into an epoxy network has been considered in detail, specifically, to investigate fundamental molecular dynamic effects.

In the area of dielectrics, the functional groups present in a material can have a marked effect on electrical properties. In the case of epoxy resins, Nguyen et al. [14] considered the effect of variations in stoichiometry on alternating current AC breakdown in anhydride-cured epoxies and showed that systems cured with an excess of hardener were characterized by a reduction in breakdown strength. Comparable effects have also been reported in the case of amine-cured systems [15], where residual epoxides had little effect on either direct current DC charge transport or DC breakdown, but residual amines markedly increased the conductivity. While such results are noteworthy, it is important to point out that in both these investigations the electrical effects described were obtained by displacing the reaction stoichiometry away from the ideal. As such, it is not possible to unambiguously deconvolute the role of retained functional groups from the influence of concomitant changes in the network structure, free volume, etc. Nevertheless, such work does lead to the intriguing corollary that if it were possible adversely to affect technologically critical macroscopic electrical properties through the inclusion of certain moieties within the network structure of epoxy resins, it may be possible to induce positive effects through the judicious choice of function group; a strategy we have termed functional network modification (FNM).

In the study reported here, we set out to establish an understanding of the effect of curing mechanisms on some key electrical parameters in a range of epoxy network systems in which the network architecture had been varied in a systematic way. For this, reference (unmodified) DGEBA epoxy resin samples cured using amine and anhydride hardeners were produced and contrasted with systems where a fraction of the epoxy resin monomer was substituted by an alternative reactive functional network modifier (FNM) which, in this case, contained both terminal epoxide groups and an unreactive group. In this way, the network structure could be modified in a controlled way and the influence of these changes on molecular dynamics be explored, through dielectric relaxation processes. In addition, the impact of changes in the network architecture on charge transport is also considered.

## 2. Experimental

### 2.1. Materials

**Figure 3:**
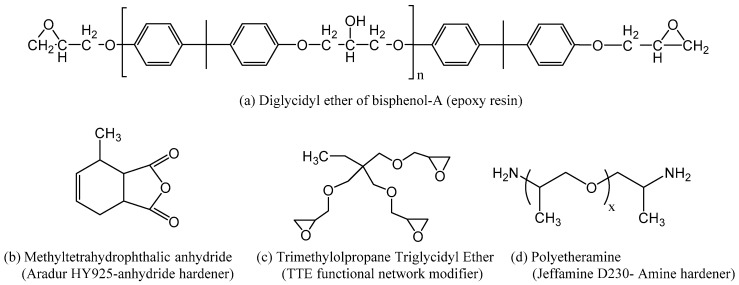
Molecular structure of: (**a**) the DGEBA epoxy resin, n = ~0.026 for DER 332; (**b**) the anhydride hardener, methyl-tetra-hydro phthalic anhydride (MTHPA); (**c**) the functional network modifier, triglycidyl ether (TTE); (**d**) the amine hardener (Jeffamine D-230: x = ~2.5).

The epoxy resin chosen for use here is a high purity diglycidyl ether of bisphenol-A based epoxy, commercially known as DER 332. This has an epoxide equivalent molar mass of 172–176 g·mol^−1^, and was obtained from Sigma Aldrich (Sigma-Aldrich, Inc., Cambridge, UK). Samples were prepared from this resin using either an amine or an anhydride hardener. The poly-ether-amine based hardener, commercially known as Jeffamin D-230, has a hydrogen equivalent molar mass of 60 g·mol^−1^ and was obtained from Huntsman (Huntsman International LLC, Llanelli, UK). The anhydride-based hardener was methyl-tetra-hydro phthalic anhydride (MTHPA), Aradur HY 925, also obtained from Huntsman. In addition to the resin and the two hardeners, the network structure of the epoxy resin was modified using a multi-epoxy terminated functional network modifier (FNM). The FNM used here was trimethylolpropane triglycidyl ether (TTE), which features three terminal epoxide groups within its chemical structure and has an epoxide equivalent molar mass of 100.79 g·mol^−1^ (obtained from Sigma Aldrich (Sigma-Aldrich, Inc., Cambridge, UK). The structure of all the molecules used in this study is shown in Figure 3.

### 2.2. Sample Preparation

Two sets of samples were manufactured for this study: amine-cured; and anhydride-cured. In all cases, the theoretical stoichiometric ratio of resin to hardener was used, 1000:344 parts by weight of resin to hardener for the amine-cured reference samples; 1000:970 parts by weight of resin to hardener for the anhydride-cured reference samples. These values are consistent with manufacturers’ recommendations. Modified samples were produced such that 1, 4, 10 and 30 mol % of the epoxide groups in the system were supplied from the FNM. In all cases, the required mass of resin, hardener and FNM was calculated so as to maintain the same theoretical stoichiometry as in the relevant reference system.

For sample manufacture, the resin was first preheated at 50 °C for 60 min, to reduce its viscosity. Then, the required mass of the three components (resin, FNM and hardener) was weighted out, and the epoxy and, where required, the FNM were mixed together for 5 min, using a magnetic stirrer. Next, the hardener was added and the complete formulation mixed for a further 5 min before being degassed. Following degassing, samples of the required thickness were cast in pre-assembled steel molds, which were then placed in a fan oven for curing. The amine hardened samples were cured at 80 °C for 2 h, then post-cured at 125 °C for 3 h, based upon a prior detailed study [16]. The anhydride-hardened systems were cured, initially at 90 °C for 2 h, then at 150 °C for 4 h, as recommended by the manufacturer. Previously, we have reported on the effect of FNMs on thermal stability [17]; the above protocol does not approach the onset of decomposition for any of the systems. The different formulations are hereafter referred to as XTTE or XATTE, where X represents the molar percentage of epoxide groups in the system that was derived from the FNM, A signifies anhydride curing and TTE indicates the appropriate FNM.

Samples for electrical characterization were 200 µm in thickness and, after production, were stored in vacuum until needed, whereupon gold electrodes were sputtered onto both surfaces, to improve the electrical contact between the sample and the measurement electrodes.

### 2.3. Characterization

Fourier-transform infrared (FTIR) spectra were acquired using an ATR-FTIR iD7 Nicolet iS5 system, from Thermo-Scientific (Thermo Fisher Scientific Inc., Winsford, UK). A background check was first conducted before collecting the spectrum of each sample. The resulting raw FTIR data were then normalized using the standard normal variate method (SNV).

Differential scanning calorimetry (DSC) was used to determine the glass transition temperature, *T*_g_, of the reference and modified epoxy resin samples. For this, a Perkin Elmer DSC7 instrument (PerkinElmer Inc., Seer Green, UK) was used, which was routinely calibrated using high purity indium. All temperature scans involved three steps: heating, cooling and a second heating, to erase the thermal history of each specimen [18]. A heating/cooling rate of 10 °C/min was used throughout. All the data reported here were obtained from the second heating scan; three repeats were conducted for each material formulation.

The molecular dynamics within the various systems were further investigated using dielectric spectroscopy. Measurements of the dielectric response were recorded using a Schlumberger SI 1260 impedance/phase gain analyzer (Solartron Group Ltd., Hampshire, UK), which was connected to a Solartron 1296 dielectric interface system (Solartron Group Ltd., Hampshire, UK). In addition, a Lake Shore 332 temperature controller was used to control the temperature of a Janis Research STVP-200-XG system cryostat (Janis Research Company LLC, Woburn, MA, USA). An AC voltage of amplitude 1 Vrms was applied and the dielectric response was recorded as a function of frequency from 1 MHz to 0.1 Hz at temperatures from −160 to 180 °C. For the low temperature measurements, the test-cell was filled with helium gas, which was cooled using liquid nitrogen but maintained at atmospheric pressure throughout. The manufacturer reports that the accuracy of the equipment is better than 0.2%, a claim verified using a fused silica reference sample, according to the method discussed in [19]. The SMaRT software, provided by the manufacturer, was used to collect the data.

The electrical conductivity of the samples was determined using a system designed and built in-house, which is described in detail elsewhere [20]. In this, the sample was placed between two parallel electrodes, 50 mm in diameter, the required voltage was applied and the resulting current was recorded as a function of time using a Keithley 6487 picoammeter (Keithley Instruments Inc., Cleveland, OH, USA).

## 3. Results and Discussion

### 3.1. FTIR Spectroscopy

**Figure 4:**
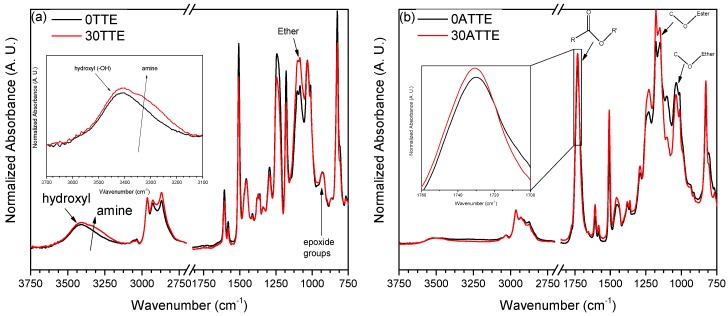
The Fourier-transform infrared (FTIR) spectra of reference and modified epoxy resin systems (**a**) amine-cured and (**b**) anhydride-cured DGEBA resin.

Normalised FTIR spectra obtained for the amine- and anhydride-cured DGEBA resin are shown in Figure 4. Since the differences between the spectra of the reference system and those containing low TTE contents are not readily visible, only data obtained from 0TTE, 0ATTE, 30TTE and 30ATTE are included here, for the sake of brevity.

Figure 4a shows data obtained from the amine-cured systems. These spectra contain four spectral regions of principal interest, namely, around 3400, 3300, 1100 and 915 cm^−1^, which are associated with hydroxyl, amine, ether and epoxide groups, respectively [21,22,23,24,25,26]. Comparison of FTIR data in the 3000 cm^−1^ region obtained from the reference system and 30TTE (see inset in Figure 4a) indicates a slight increase in the amine absorbance region in the latter system. While variations in IR peak intensities must be interpreted with care, especially when the data are derived from solid material, nevertheless, this change suggests that the inclusion of the TTE results in an increased probability of unreacted secondary amine groups being retained within the system. Since the initial stoichiometry was chosen to ensure equivalent numbers of amine hydrogens and epoxide groups, at first sight, this implies a commensurate increase in unreacted epoxide groups in 30TTE, which is not the case from the epoxy peak at 915 cm^−1^, which is identical in both systems. 

Rather, the increased relative absorbance in the range from 1060 to 1150 cm^−1^ seen in 30TTE implies a a system with a higher ether content. While the TTE and epoxy resin reactants both contain ether groups, since n = 0.026 for DER 332, to a very good approximation, both contain one ether group per epoxide, such that substituting resin epoxide groups with TTE epoxide groups should have a negligible effect on the concentration of ether linkages present in the system, prior to curing. As such, the increased ether absorption seen in 30TTE in Figure 4a is likely to be a consequence of the imposed curing protocol, presumably, etherification/homopolymerization at the elevated temperature used for post-curing. This behavior is similar to the reactions caused by post-curing of epoxy resin systems reported by Vryonis et al. [27]. In the case of the anhydride-cured systems, consider the hydroxyl-related absorbance region around 3400 cm^−1^ together with the peak at 1680 cm^−1^ and the region from 1250 to 1150 cm^−1^ that are associated with ester groups [24,25,26]. As described above, OH groups are both consumed and generated during anhydride curing through esterification. Therefore, as indicated above, it is not straightforward to interpret the observation that the OH absorption in 30ATTE appears somewhat greater than in the reference system, particularly in view of the spectral normalization procedure used here. However, Figure 4b also suggests a small increase in the strength of both the ester peak around 1680 cm^−1^ on adding the FNM and the related absorption between 1250 and 1150 cm^−1^, which is associated with stretching of the C–O groups of esters [28,29,30]. In parallel, the band between 1150 and 1060 cm^−1^, which is related to the stretch of C–O of ethers [21,31], seems reduced somewhat in 30ATTE. In toto, the above data therefore suggest that the inclusion of TTE affects the relative probability of crosslinking by esterification and etherification/homopolymerization and, in this way, influences the precise network topology that forms during anhydride curing.

### 3.2. Differential Scanning Calorimetry

**Figure 5:**
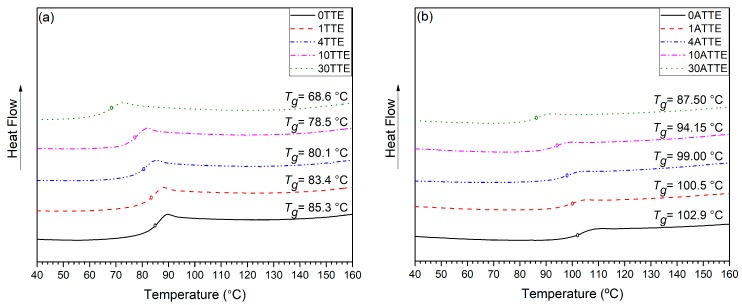
The differential scanning calorimetry of reference and modified epoxy resin systems (**a**) amine-cured and (**b**) anhydride-cured DGEBA resin.

*T*_g_-related data obtained by DSC are presented in Figure 5 and listed in Table 1. Evidently, introduction of TTE results in a progressive and equivalent reduction in *T*_g_ in both the amine- and anhydride-cured systems with, in all cases, the *T*_g_ of the latter being some 20 °C higher than that for the same composition cured using the amine.

*T*_g_ is often considered to be closely related to the free volume in the system [3,18,32,33] and, consequently, the data presented in Table 1 could be related to changes in this factor that result from (a) the choice of hardener; and (b) the incorporation of the TTE functional network modifier. However, comparison of the molecular structure of TTE, which contains an unreactive methyl group, with the behavior of FNMs containing long alkyl chains reported in a previous work [34] suggests that the direct influence of TTE on free volume should be small, implying in turn, that alternative factors are at play. Indeed, Morgan et al. [35] reported a local minimum in the macroscopic density at maximum *T*_g_, which they associated with geometric constraints imposed on segmental packing by the crosslinks, while Alhabil et al. [36] suggested that the *T*_g_ of epoxy resin systems is greatly influenced by the precise network topology that forms. 

In the case of amine curing, epoxide groups react preferentially with primary amines, such that unreacted amine hydrogens will, in the form of secondary amines, tend to be located between network nodes, while unreacted epoxides will be present in the form of chain ends. In the sample preparation protocol used here, post-curing was conducted at a constant temperature and, as such, as the TTE content is increased, so the difference between *T*_g_ and the post curing temperature increases. This may then serve to promote etherification/homopolymerization reactions of epoxide chain ends with hydroxyl groups, so leading to the increased strength of the ether absorbance peak in the 30TTE FTIR data in Figure 4a. The net consequences of residual secondary amines in systems containing TTE will be an increase in the average contour length between network nodes and increased chain mobility, which aligns with the observed reduction in *T*_g_. Also, Neville and Lee [1] reported that the presence of aromatic groups in the structure can influence *T*_g_, which may be a further contributing factor. Replacement of epoxy groups from DGEBA with epoxy groups from TTE will reduce the overall aromatic content of the system. 

In the case of anhydride crosslinking, it is difficult to suggest comparable possible chemical pathways relating the chemical changes revealed by FTIR to changes in *T*_g_. However, the observed differences in the ether and ester regions of the FTIR spectra clearly indicate that varying the chemical composition materially affects the crosslinking reactions that occur, giving rise to different network structures. 

### 3.3. Dielectric Spectroscopy

**Figure 6:**
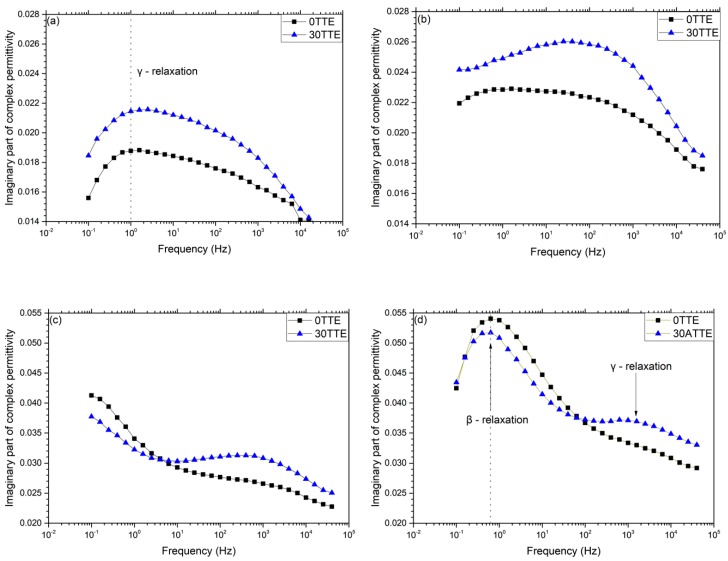
Imaginary part of complex permittivity of reference and modified amine-cured resins at: (**a**) −130 °C; (**b**) −110 °C; (**c**) −90 °C; (**d**) −70 °C. Note: the increase in ordinate scale in (**c**) and (**d**) necessary to present the β relaxation.

**Figure 7:**
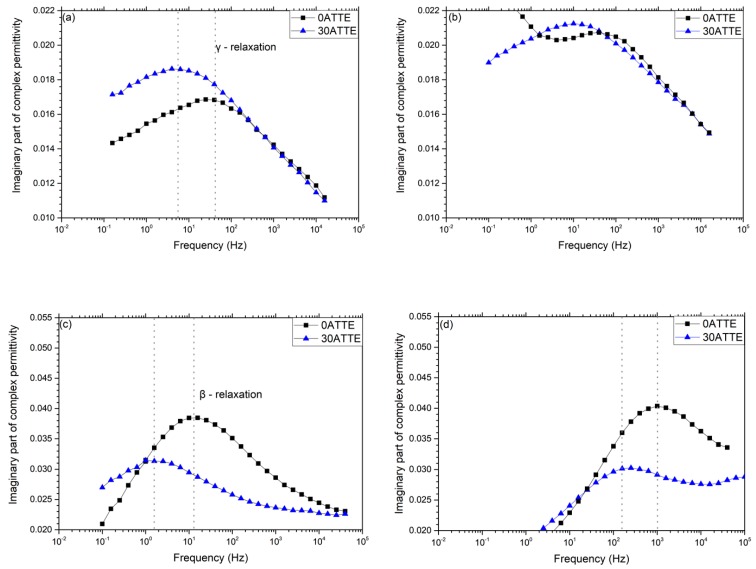
Imaginary part of complex permittivity of reference and modified amine-cured resins at: (**a**) −130 °C; (**b**) −110 °C; (**c**) −70 °C; (**d**) −30 °C. Note: the increase in ordinate scale in (**c**) and (**d**) necessary to present the β relaxation.

The relationships between chemical composition, reaction pathway and network topology were further examined by considering the molecular dynamics of the various systems, as revealed by dielectric spectroscopy. The imaginary part of the complex relative permittivity, *ε”*, obtained at selected temperatures between −130 and −30 °C for the two reference systems is compared with equivalent data obtained from 30TTE and 30ATTE in Figure 6 and Figure 7 respectively. Although data were obtained at 10 °C intervals from −160 to 180 °C, only results at the indicated temperatures are presented here, for the sake of brevity. The specific temperature ranges were chosen based on the work of Vryonis et al. [27].

Consider, first, the effect of including TTE on the γ relaxation of the amine-cured systems, shown in Figure 6. In both the reference resin and 30TTE, the γ relaxation manifest itself as a broad peak in *ε”* spanning the frequency range from 10^−1^ to 10^4^ Hz, which suggests that it stems from the motion of a number of different dipolar species and/or the existence of differently constraining environments. Comparison of the data obtained from the two systems demonstrates that inclusion of TTE results in an increase in the strength of the γ relaxation, particularly at frequencies below about 100 Hz. The epoxy γ relaxation has previously been discussed in terms of numerous different molecular species, including terminal dipolar moieties such as unreacted epoxides and amine groups [18]. Such an attribution aligns with the increased FTIR absorbance that is evident in the amine region of 30TTE in Figure 4. However, as described above, these amine groups are likely to be present, largely, as secondary amines incorporated into the network between nodes, rather than being present as terminal, primary amines. Indeed, Hassan et al. [37] have proposed that the epoxy γ relaxation is related to the motion of amino-diphenyl groups, while Jilani et al. [38] suggested that ether linkages can be important in facilitating the motion of interposed groups. Although such effects are broadly consistent with the increase in *ε”* seen in 30TTE, in formulating 30TTE, epoxide groups from the resin were substituted by epoxide groups from the FNM and, as a consequence, the concentration of aromatic structures in 30TTE would be reduced compared with 0TTE. As such, we suggest that the observed γ relaxations are a consequence of three factors: (a) unreacted dipolar end groups; (b) the motion of moieties associated with secondary amines located between network nodes; and (c) modified molecular topologies and reduced aromatic content.

Figure 6d compares imaginary relative permittivity data acquired from the reference resin and 30TTE at −70 °C. Consider, first, the region from 0.1 to 10 Hz, which contains a feature that we ascribe to the β relaxation. Although this peak appears comparable in both systems, its strength is somewhat reduced in 30TTE compared with the reference epoxy, a result that is consistent with the notion that the β relaxation in amine-cured epoxies stems from –CH_2_–CH(OH)–CH_2_–O– (hydroxyether) moieties that are formed as a result of the epoxide/amine crosslinking reaction [18,39]. This interpretation aligns with the increased amine and ether absorption seen in 30TTE by FTIR, which implies a reduced hydroxyether content compared with 0TTE. The molecular origin of the second much weaker dielectric process in 30TTE at frequencies above 100 Hz is, however, less clear but from consideration of the complete data set shown in Figure 6, we suggest that this feature is related to the γ relaxation. However, comparison of Figure 6a,b reveals a marked change in peak shape on increasing the temperature from −130 to −110 °C, particularly in the case of 30TTE, which may be indicative of a number of different processes/moieties being involved. Mikolajczak et al. [40] suggested that the epoxy γ and β relaxations relate, respectively, to regions that differ with respect to the local molecular mobility, such that each region is characterised by an activation energy that is determined by the corresponding environment. Elsewhere, Hassan et al. [37] reported on a DGEBA resin crosslinked using two related diaminodiphenyl sulfone crosslinkers and interpreted their observations as a bifurcation in the α relaxation that was related to variations in the contour length of sub-chains between network nodes. Accordingly, a plausible explanation for the different γ relaxation behavior seen in 0TTE and 30TTE concerns the relative contributions in each of the different dipolar species described above moving within different molecular environments, which could reasonably be expected to be characterised by different activation energies and therefore exhibit different temperature dependencies. Indeed, in Figure 6c, a weak γ relaxation is still visible in the dielectric response of 0TTE but at an apparently higher frequency than in 30TTE. These issues will be addressed more quantitatively elsewhere [41].

Figure 7a shows the imaginary part of the complex permittivity obtained at −130 °C from the reference and TTE-modified anhydride-cured systems. The γ relaxation of the reference 0ATTE appears as a pronounced feature with a peak maximum at ~40 Hz. At higher frequencies, 0TTE and 30TTE exhibit identical behavior, while the losses associated with the latter system are markedly increased at lower frequencies. Consequently, the maximum in *ε”* in 30ATTE occurs at ~5 Hz. Increasing the temperature to −110 °C results in the displacement of the γ relaxation of both systems to higher temperatures and, in the case of 0ATTE, increasing losses below 1 Hz; the data obtained at −70 °C (see Figure 7c) indicate that this should be associated with the β relaxation, which we will consider first, before returning to the γ relaxation.

While there is a strong consensus in the published literature concerning the principal molecular process that gives rise to the β relaxation in amine-cured epoxies, the equivalent topic in anhydride-cured systems has attracted little attention. Nevertheless, Cuddihy and Moacanin [42] and Ochi et al. [39,43] have contrasted the impact of a number of different anhydrides on network evolution in a DGEBA-type epoxy resin and highlighted the importance of the diester segments formed during anhydride curing in facilitating the motion of the interposed anhydride residue. This occurs to a degree that is determined by the molecular structure of the anhydride, combined with local steric factors. We similarly propose that the β relaxation in anhydride-cured systems is related to motion related to diester bridges, such that the strength of this process is again closely related to the extent of crosslinking. However, the FTIR data shown in Figure 4b also reveal differences in the crosslinking process between 0ATTE and 30ATTE. Specifically, the peaks around 1680 cm^−1^ and the region from 1250 to 1150 cm^−1^, both of which are derived from ester groups, are stronger in 30ATTE than in 0ATTE. Conversely, the ether-related absorption at 1150 to 1060 cm^−1^ is stronger in 0ATTE than in 30ATTE. In line with the argument present previously, the presence of increased ether linkages in 0ATTE must result from the reaction of epoxide groups with hydroxyls present in the system. Indeed, comparison of the molecular structures of DGEBA and TTE reveals that, while the former contains potentially reactive OH groups, the latter does not. As such, the concentration of hydroxyl groups in the reactants in 0ATTE is higher than in 30ATTE, suggesting that the increased ether absorption seen in the FTIR spectrum of the former system may result from increased homopolymerization. Consequently, the higher glass transition temperature of 0ATTE correlates well with the increased strength of the β relaxation of 0TTE in Figure 7c,d, combined with FTIR evidence of additional crosslinking through homopolymerization in this system.

A corollary of epoxide consumption through additional homopolymerization concerns the unreacted species that must then be retained within the network. Since both 0ATTE and 30ATTE were produced using the same stoichiometric ratio of epoxy to anhydride, a relative increase in 0TTE in the consumption of epoxide groups through etherification/homopolymerization, compared with consumption through their reaction with the anhydride hardener (esterification), implies a shift in the balance of the related species retained in the system. While it is possible that some unreacted anhydride, DGEBA and TTE molecules may be trapped within the vitrified network, it is more likely that the bulk of the retained unreacted functionality will exist as chain ends terminated with epoxide groups or anhydride residues. As such, we suggest that the differing strength of the γ relaxation seen in the two systems is related to variations in the concentration of chain end groups in each system, an interpretation that parallels commensurate published work on amine-cured epoxies.

The analysis presented above considers the observed dielectric relaxations in terms of the chemical variations revealed by FTIR. While it is possible in some circumstances to propose associations between chemical species and dielectric effects [37,39,43,44], we suggest that the results presented above also highlight the importance not just of the dipolar units involved, but also their local environment in determining the observed dielectric response. In the case of crosslinked networks such as epoxy resins, the inherent range of structures that may form makes detailed analysis difficult; although molecular dynamics simulations of epoxy networks have been reported [45,46,47,48], we are not aware of any attempts to extend such an approach to consider dielectric factors. Nevertheless, comparable issues have been considered in some detail in a number of thermoplastic polymer systems that exhibit a high glass transition temperature and which contain both ether linkages and aromatic rings. Such systems, therefore, have certain parallels with those reported here, albeit that their thermoplastic nature renders them more amenable to detailed analysis. 

The influence of thermal ageing on molecular relaxation processes has been reported for poly(ethylene terephthalate) (PET), poly(ethylene naphthalate) (PEN) and a series of poly-(ethylene terephthalate-co-ethylene naphthalate) (PETN) copolymers [49]. In this work, the β relaxation in all the systems studied was found to be broad, which was interpreted as reflecting the motion of diploes within differing local environments as a consequence of the non-uniformity of the glassy state. Specifically, it was suggested that localized chain motions associated with the β relaxation process contribute to physical ageing, such that a more densely packed structure with diminished mobility results. In these systems, increased ordering was suggested to occur through chain segment alignment involving trans-ethylene glycol conformations, coplanar CO units and parallelization of benzene rings. Verot et al. [50] examined molecular relaxation processes in poly(aryl ether ether ketone) (PEEK) and related the observed double β relaxation to the movement of two classes of diphenyl ketone (DPK) units, through gear rotation motions. The first was related to the motion of DPK units remote from one another in a single molecule, the second being associated with the motion of DPK units in closer proximity to one another and present as “entangled or ball-forming” conformations. This paper further discussed the γ relaxation in PEEK, indicating that, at low temperatures, this could be related to highly localized wagging of polar bridges, which progressively transforms into β motions at higher temperatures. Elsewhere [51], such processes were studied in PEEK, using a combination of electrical techniques, where the γ and β relaxations were related to the localized rotational mobility of phenylene rings and the displacement of conformational defects, respectively. Arrhenius analysis of the γ, β and α relaxations showed a progressive increase in the associated activation enthalpy and entropy, which was taken to indicate an increase in the size of the domains where cooperative mobility takes place on moving from the γ to the β to the α relaxation. As such, it is perhaps inappropriate to associate different dielectric relaxations exclusively with different dipolar species but, rather, to consider them as originating from the coupling of the applied field via dipolar units through to larger molecular units within the system. Indeed, Rault [52] considered the α relaxation as arising from the simultaneous occurrence of a number of β-type processes, thereby explicitly stating the inter-related nature of molecular relaxations. 

In summary, the dielectric response of epoxy systems has been reported by many workers and various different molecular origins for the different observed relaxations has been proposed. Although the results derived from the systematically varied range of systems considered here do not permit us unambiguously to provide a total molecular interpretation for all the effects we report, we do believe that the following is warranted. Specifically, we suggest that coupling between dipolar units and the local network structure is critical and that the γ, β and α relaxations—the last of which will be considered elsewhere [41]—relate to an increase in the size of the domains within which cooperative motion takes place. As such, we primarily relate the γ relaxation to small molecular units including unreacted epoxide chain ends, anhydride-residue chain ends and secondary amines. The β relaxation in amine-cured systems involves the motion of chain sequences, notable –CH_2_–CH(OH) –CH_2_–O– in amine-cured system and diester segments formed during anhydride curing. That is, in both cases, the chain sequences formed during curing, although chemically different, appear to be closely related to the β relaxation. 

### 3.4. Electrical Conductivity

**Figure 8:**
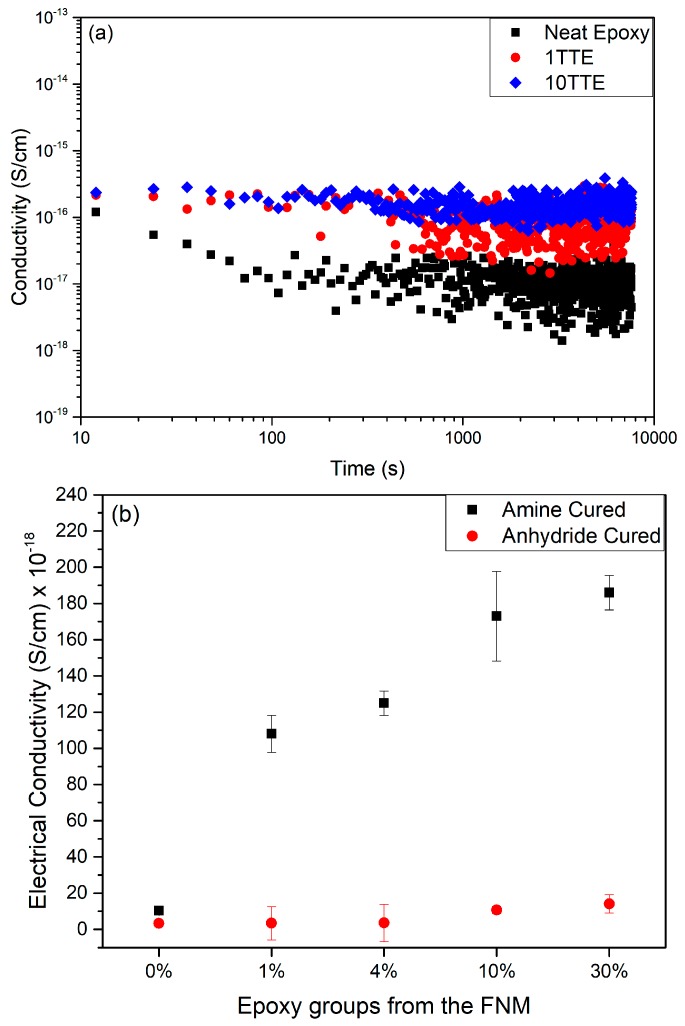
Electrical conductivity data presented in (**a**) as a function of time for the three indicated amine-cured systems and, in (**b**), as the dependence of electrical conductivity on material formulation.

Conductivity data obtained from the various systems considered here are presented in Figure 8. Figure 8a contains results obtained from three amine-cured systems: the base resin, 1TTE and 10TTE. These data sets do not overly overlap with one another and, therefore, reveal clearly the typical variation of conductivity with time. From these, it would appear that, with the possible exception of data acquired over the first 100 s, the current flowing through each system is constant. To explore the veracity of this statement objectively, a statistical analysis was undertaken; if conductivity were time independent, then plotting this parameter against time will conform to a straight line with zero gradient. Such an analysis provided no evidence of significant variations in conductivity with time, such that the behavior of each formulation can reasonably be represented by a DC conductivity value. The effect of the FNM on the DC conductivity of the amine and anhydride cured resins is therefore presented in Figure 8b. From this, it is evident that 0TTE and 0ATTE are characterised by comparable conductivity values, ~ 10.3 ± 0.5 × 10^−18^ and 3.4 ± 1.3 × 10^−18^ S/cm respectively, indicating that the choice of hardener does not, *per se*, have a major effect on charge transport under the conditions used here. In the case of the systems subjected to anhydride curing, substitution of DGEBA by TTE has a minor effect, in that only at the highest concentrations of TTE is a small (from 3.37 × 10^−18^ to 14.1 × 10^−18^ S/cm) increase in conductivity observed. In contrast, the XTTE sample set is characterised by a marked monotonic increase in conductivity with TTE content. While a precise mechanistic interpretation of the origin of charge transport in cpmplex systems such as the one considered here is extremely difficult, the effects that are evident in Figure 8 can be discussed in terms of four factors:

- Moieties explicitly introduced through the inclusion of the FNM;
- Variations in free volume in the various formulations;
- Changes in network dynamics that result from changes in network topology;
- The presence of specific unreacted functional groups from the resin and hardener.

The FNM used in this study features three epoxide groups within its chemical structure and, as such, takes part in the curing process, leading to the introduction of methyl “branches” Figure into the molecular network. Although it has previously been reported that the inclusion of alkyl chains can impact the electrical behavior of an epoxy resin [53], the observation that the electrical conductivity of the anhydride cured systems is largely invariant of TTE content demonstrates that the presence of the methyl groups is not, in itself, responsible for the increased conductivity seen in the amine-cured systems. Theoretical calculations of the behavior of an excess electron in polyethylene has shown that localized regions of free volume constitute low energy states for electrons and that such electron states can become delocalized, at least over the length scales considered within the simulations [54,55]. However, in view of the commensurate variation in *T*_g_ with composition seen on use of the two different curing chemistries, it is difficult to ascribe the pronounced increase in conductivity seen in only the amine-cured samples on introducing TTE to variations in free volume. Furthermore, theoretical studies of the dynamical properties of excess electrons within amorphous regions of polyethylene [56] have indicated that these become self-trapped as localized polarons. The estimated value of self-trapping energy was found to be small, leading to the proposition of charge transport through a mechanism of phonon assisted hopping in this system. The dielectric data presented above demonstrate variations in segmental molecular dynamics with TTE content in both amine- and anhydride-cured epoxy systems such that, again, the invariance in DC conductivity with composition seen in the former material set indicates that composition-related variations in molecular dynamics is unlikely to be the source of the increase in DC conductivity (from 10.3 × 10^−18^ S/cm for the 0TTE to 186 × 10^−18^ S/cm for the 30TTE) seen on comparing 0TTE and 30TTE.

Finally, a number of studies [1,57] have reported that charge transport through an amine-cured resin network is influenced by the linkages generated during the reactions of the primary and secondary amines, while Alhabill et al. reported increased DC conductivity in systems where the stoichiometry had been offset to an excess of amine [15]. From the FTIR and dielectric spectroscopy data presented above, it is evident that TTE-modified amine-cured systems contain an increased number of unreacted secondary amine groups. As such, we believe that the work reported here clearly demonstrates that the charge transport behavior presented in Figure 8 is indeed related to the functional groups present in each of the systems. This conclusion therefore is consistent with the theoretical studies of Meunier and Quirke [58], whereby the presence of molecules with electroactive behavior (amines in the system considered in our study) serves to alter the local density of states which, in turn, affects charge transport through the system.

## 4. Conclusions

A range of amine- and anhydride-cured epoxy resin systems have been produced in which the network structure has been systematically varied through the substitution of epoxide groups from the DGEBA with epoxide groups from an additional component, which we have termed a functional network modifier. Inclusion of the FNM modifies the chemical reactions that occur during curing. Specifically, we suggest that during amine-curing of systems containing the FNM, increased homopolymerization occurs, which serves to consume epoxide groups. Since all systems were formulated using equivalent epoxide/hardener ratios, this leaves an increased concentration of secondary amine groups within the network, which increases the average contour length between network nodes. While the nature of anhydride-curing does not permit a comparable interpretation, the FTIR data indicate that the inclusion of the TTE affects the relative probability of crosslinking by esterification and etherification/homopolymerization and, in this way, modifies the network topology that develops. In both sets of systems, introduction of the TTE FNM acts to reduce the glass transition temperature and influence the dielectric molecular relaxation behavior. Consideration of FTIR, *T*_g_ and dielectric data in concert leads us to suggest that in both amine- and anhydride cured epoxies, the γ relaxation is related to the local motion of small dipolar units: epoxide chain ends; anhydride-residue chain ends; secondary amines. The β relaxation in both systems is related to larger structural units and is closely associated with crosslinking: that is, hydroxyether units in amine-cured systems and diester segments in anhydride-cured materials.

A primary aim of the study reported here was to test rigorously the hypothesis that charge transport behavior through an epoxy network could be modified through grafting appropriate functional groups into the network. Although previous work was suggestive of this, the rather crude methodology of displacing the curing stoichiometry away from the ideal rendered the results inconclusive. Here, this has been overcome through the use of the FNM strategy in conjunction with the two different curing chemistries. Consideration of the effect of both approaches in concert enables us to demonstrate that the variation in conductivity seen in the amine-cured systems on introducing the FNM is not related to the methyl groups introduced as a consequence, systematic variations in free volume which constitute low energy excess electron states, or changes in molecular dynamics that affect charge transport through a mechanism of phonon assisted hopping. Rather, we believe that the work here demonstrates clearly the critical role played by the functional groups that are retained within the epoxy network. Here, we specifically relate the observed increase in conduction to increased amine contents; while this may be technologically desirable in some applications (e.g., as a means of dispersing accumulated space charge in high voltage DC power transmission applications), we feel that the significance of these results is, rather, as a proof of concept that paves the way to the development of material systems where the retained functionality is actively designed to provide the required electrical response.

## Figures and Tables

**Table 1:** Differential scanning calorimetry (DSC) measurements for reference and modified epoxy systems.

Sample	*T*_g_ (°C) Amine-Cured (±2 °C)	*T*_g_ (°C) Anhydride-Cured (±2 °C)
Reference	85.3	102.9
1%	83.4	100.5
4%	80.1	99.0
10%	78.5	94.15
30%	68.6	87.5

## Appendix A

The following figure presents a comparison between etherification and homopolymerization for amine-cured systems.
